# Acupuncture Anxiolytic Effects on Physiological and Psychological Assessments for a Clinical Trial

**DOI:** 10.1155/2016/4016952

**Published:** 2016-04-04

**Authors:** Monir Shayestehfar, Tohid Seif-Barghi, Sahar Zarei, Amir Mehran

**Affiliations:** ^1^Neuroscience Institute, Tehran University of Medical Sciences, Tehran, Iran; ^2^Sports Medicine Research Center, Tehran University of Medical Sciences, Tehran, Iran

## Abstract

In a randomized controlled trial we examined the effect of acupuncture on anxiety of the adolescent football players prior to the competition using psychological and physiological markers. A total of 45 athletes were equally allocated to either acupuncture group, sham group, or wait-list control group. Thereafter, all participants were asked to complete an anxiety questionnaire before and after the intervention. Their heart rate and skin conductance were also examined before and after the intervention. The results of ANOVA on posttest scores showed that acupuncture had a significant effect on cognitive anxiety (*p* = 0.001) and somatic anxiety (*p* < 0.001) but not on self-confidence (*p* > 0.05). Furthermore, the results showed that acupuncture significantly decreased the skin conductance in acupuncture group compared to sham group (*p* = 0.006) and wait-list control group (*p* < 0.001). In conclusion, the results suggested that acupuncture has the capacity to decrease cognitive anxiety and somatic anxiety prior to competition in adolescent athletes, while this was accompanied by significant physiological changes. This trial is registered with IRCT138904074264N1 (IRCT is a Primary Registry in the WHO Registry Network).

## 1. Introduction

Athletes face a wide range of stressors due to challenging nature of the competitive environment. When these competitive demands are beyond the athlete's coping ability, they may lead to competitive anxiety. This is a specific negative emotion that may result in an unexpected error or freeze the athlete to the point where he/she is no longer able to effectively function [1]. Although a certain level of competitive anxiety may increase performance, many athletes with anxiety experience uncontrolled negative feelings and cognitions which in turn can have overwhelming effects on their performance [2, 3]. Competitive anxiety may also result in a decrease in perceptions of competence and enjoyment as well as an increase in injury incidence or injury severity [4, 5]. Indeed competitive anxiety includes state (acute) and trait (chronic) dimensions. Trait anxiety refers to the way that individuals feel threatened or discomforted during typical situations and the tendency to respond to this nonthreatening condition [6]. However, state anxiety refers to a temporary and subjective experience of unpleasant feeling of tension, fear, and nervousness in a particular situation which is usually accompanied by arousal of the autonomic nervous system. These two types of anxiety will respond to interventions differently [7–10] and in current study we intended the type that is close to a competition, namely, state anxiety.

To manage the competitive anxiety, various cognitive and behavioral interventions such as relaxation techniques, hypnosis, and a large treatment category named cognitive behavioral treatments (CBTs) are frequently used by athletes [11, 12]. For instance, CBT includes strategies for cognitive restructuring (i.e., replacing negative thoughts with positive accounts) in addition to behavioral strategies (e.g., relaxation). Decreases in negative feeling, fatigue, and levels of the cortisol are proposed as mechanisms underlying positive effects of CBT on athletes' anxiety [13]. However, these methods have not been without limitations. Such interventions usually need a persistent team work with a therapist to manage the anxiety response and consequently take a lot of time for the athlete. Furthermore, methods with cognitive reconstruction phase may cause the athlete to become more anxious or emotionally uncomfortable in initial periods. However, there were alternative interventions (e.g., acupuncture) which were less intrusive, less time-consuming, and more economic and hence were better candidates for use in athletes' environment [14]. Recently researchers administered acupuncture as a treatment that could successfully reduce anxiety or depressive symptoms in clinical populations [15, 16]. Acupuncture was also suggested to reduce the anxiety of adolescents in high pressure situations such as surgery, academic examination, or sport competition [17, 18]. Primary results from a very recent pilot study of effectiveness of acupuncture in reducing competitive state anxiety of track and field athletes have confirmed the safety of acupuncture and supported its use in athletes [14].

Evidence on regulation of competitive anxiety indicated the role of hypothalamus-pituitary-adrenal (HPA) axis hyperresponsiveness to competition demands. For example, Filaire et al. showed an increase in level of cortisol in female athletes prior to a competition compared to a control group [19]. On the other hand, Eshkevari et al. revealed that acupuncture shows its specific effects on anxiety by reducing acute stress-induced elevations in the HPA hormones (i.e., ACTH, cortisol, and CRH) [20, 21]. Animal neuroimaging studies also showed that acupuncture can reduce anxiety-like behaviors by blocking the neuropeptide Y pathway in amygdala [22]. Furthermore, acupuncture showed strong effects on endogenous opioids in the different brain regions (e.g., amygdala), which might lead to a decrease in context specific anxiety responses [23, 24]. Given together, since athletes experience high level of state (or acute) anxiety before the competition [9, 10], it would be expected that an acupuncture session helps athletes to decrease their competitive state anxiety [25].

However, as far as we know, there was no published study to evaluate the effect of acupuncture on athletes' competitive anxiety. In this study, we aimed to assess the effect of the acupuncture on competitive anxiety in a sample of young football players using physiological biomarkers in addition to subjective anxiety measures. We then hypothesized that acupuncture intervention could decrease the state anxiety of athletes prior to a competition event. We further hypothesized that acupuncture would help athletes to reduce the physiological responses (skin conductance and heart rate) just before the competition event.

## 2. Methods

### 2.1. Participants

Participants were 45 male football players (16–18 years old) from three different elite clubs in Tehran. Participants were randomly divided into an intervention group (*n* = 15), a sham group (*n* = 15), and a wait-list control group (*n* = 15). Participants were examined by a physician to screen for medication history, medical illnesses, and history of psychopathologies including anxiety or depressive disorders.

Inclusion criteria for the participants were being athlete aged 16 to 18, no history of acupuncture treatment in three months prior to the examination with willingness to receive acupuncture, and assignment of the informed consent. Exclusion criteria were use of any psychotropic medications including anxiolytic medications, presence of any chronic medical or mental health disorders (e.g., psychosis or depressive disorders), and occurrence of any adverse reactions to acupuncture. In primary evaluation we asked the participants to complete the informed written consent form. The ethic committee of the Tehran University of Medical Sciences approved this study.

### 2.2. Apparatus

Acupuncture group received acupuncture on relaxation and Shen Men point using clean needle after trimming the considered points of skin by alcohol for 30 minutes. Intervention was conducted in a silent place when participants were in lying position. Sham group (i.e., placebo) received sham acupuncture on the identical points considered for (true) acupuncture group using specific needles for sham. Similar to acupuncture group, sham needles were also retained for 30 minutes. An expert in acupuncture inserted the needles for all participants in acupuncture group or sham group. During primary and secondary evaluations, control group was in waiting list and did not receive any treatment.

### 2.3. Procedure

Young athletes who met the inclusion/exclusion criteria were selected by the principal researchers. Eligible participants were randomized by a computer-generated schedule to receive either one of three possible intervention sequences (acupuncture, sham, or wait-list control). A research assistant performed the randomization and allocation procedure. The athletes (in acupuncture and sham groups) and examiners were blind to the intervention allocation, but the acupuncturist was not blinded.

All the athletes were inquired to complete the same questionnaires before and after the intervention in order to examine their levels of anxiety. They also underwent identical physiological examinations (heart rate and skin conductance) before and after the intervention. To determine the appropriate time space for administrating acupuncture, previous data indicated that the anxiety symptoms show increasing levels as the competition approached (up to a few hours prior to competition) [25]; furthermore the positive effects of acupuncture on state anxiety begin 30 minutes after administration [17]. Given together, in current study, participants received intervention (acupuncture or sham) four hours before the competition event. The first questionnaire and physiological examination were administered one hour before the intervention, and secondary evaluations were performed half an hour after the intervention. To control for the effect of potential confounders on the results of physiological assessments, participants were asked not to use medicine, alcohol, or caffeine and to get a good night of rest during 24 hours prior to the assessment. Prior to the intervention, two familiarization sessions were conducted aiming to instruct acupuncture technique for athletes in acupuncture and sham groups and all assessments were accomplished by an educated examiner.

### 2.4. Assessment

Before and after each treatment session, the physiological data were obtained using ProComp Infiniti hardware from Thought Technology*™*. ProComp Infiniti is a Biofeedback system with 5 channels. Skin conductance sensor and heart rate (HR)/blood vessel pressure (BVP) sensor recorded physiological state of participants objectively by a multimodality encoder. The recorded data was transferred to the computer with fiber optic cables and was processed using Biography software. The heart rate of participants was calculated based on filtered BVP using peaks and valleys of BVP. Peaks and valleys were calculated based on the points where derivative of BVP reached zero. HR was calculated by this formula using the time between peaks: HR = 1/time. Trends of BVP signals were removed. The uniformity of rate was assured by resampling all signals at 8 samples per second. The skin conductance sensor was used to measure the conductance through the skin. The skin conductance had two small electrodes that were placed around the index and annular fingers by straps. Each of the electrodes had to be fixed in interior side of finger by finger straps. An inappreciable and little voltage was exerted across the skin to measure resistance. Skin conductance is influenced by the activity of sweat glands and the skin's pore size which are influenced by sympathetic nervous system that controls activity of sweat glands. When individuals experience anxiety an increase in sweat glands' activity will accrue which will lead to fast increase of skin's conductance. The increased activity fills gland ducts with sweat after a while, which makes increment of skin conductance impossible. After anxiety period, naturally the skin conductance will decrease because of the reabsorption of secretions. In current study, an average score of skin conductance during 10 minutes of assessment was used for analysis.

To examine psychological outcomes, the Competitive State Anxiety Inventory-2 (CSAI-2), a 27-item questionnaire including three subscales of cognitive anxiety, somatic anxiety, and self-confidence, was used (Table 3). The CSAI-2 items are rated on a 4-point Likert scale ranging from 1 (“Not at all”) to 4 (“Very much so”). All scores of the items are at face value except for item 14 which is reverse. In several studies, the CSAI-2 indicated high internal consistency over different cultures [26]. Cronbach's alpha for the current sample was 0.83.

### 2.5. Statistical Analysis

Based on changes in state anxiety scores before and after acupuncture, the sample size was estimated. With a significance level of 0.05 and power of 0.80 and a medium effect size of 0.4, a total sample size of 45 with 15 for each group was required. Variables were assessed for normality of distribution. All data were analyzed by 3 × 2 (group × time) mixed two-way ANOVA/MANOVA. When appropriate, a post hoc test was conducted. Descriptive data for general records were reported (mean ± SD). *P* values less than 0.05 were considered as statistically significant. Analyses were performed using the SPSS software version 17 for Windows (SPSS Inc., Chicago, IL, USA).

## 3. Results

### 3.1. Skin Conductance

Table 1 indicates mean scores and standard deviation of skin conductance measures. A significant main effect was found for time: *F*(1,42) = 17.629; *p* < 0.001. The interaction group × time was also significant: *F*(2,42) = 5.222; *p* = 0.009. Post hoc analysis revealed significant more decrease at the acupuncture compared to sham group (*p* = 0.04) and control group (*p* < 0.018). However, sham acupuncture showed no significant changes compared to control group.

### 3.2. Heart Rate

The means and standard deviations of heart rate at the pre- and posttest time points for each group are presented in Table 2. A significant main effect was found for time: *F*(1,42) = 7.316; *p* = 0.01. The interaction group × time was significant: *F*(2,42) = 18.940; *p* < 0.008. Post hoc analysis revealed significant more decrease at the acupuncture group compared to sham group (*p* = 0.004) but not control group (*p* > 0.05). However, heart rate changes in sham group were not different with control participants across the intervention phase.

### 3.3. Competitive State Anxiety

A mixed two-way ANOVA for cognitive anxiety or somatic anxiety or self-confidence as the dependent variable over time (i.e., pre- and posttest scores) and group as the independent variable was computed. A significant main effect for time emerged for cognitive anxiety (*F*(1,42) = 21.872; *p* < 0.001) and somatic anxiety (*F*(1,42) = 26.053; *p* < 0.001) but not self-confidence (*p* > 0.05). Further analysis of the significant interaction time × group was investigated and revealed that there is a significant interaction between time and group for cognitive anxiety (*F*(2,42) = 28.143; *p* < 0.001) and somatic anxiety (*F*(2,42) = 32.234; *p* < 0.001). Although results showed no significant changes in confidence scores between and within groups, the results of post hoc analysis showed that the acupuncture had a significant decreasing effect on cognitive anxiety compared to sham group (*p* = 0.001) and control group (*p* ≤ 0.001). Also acupuncture had a significant decreasing effect on somatic anxiety compared to sham group (*p* < 0.001) and control group (*p* < 0.001).

## 4. Discussion

In a clinical trial of forty-five subjects, administration of acupuncture prior to competition was associated with decreased level of both components of competitive anxiety (somatic and cognitive). Those participants who received acupuncture had lower activation of the skin conductance compared to control and sham groups. This finding is consistent with a study reporting that insertion of acupuncture needle is associated with alteration of skin potential and in turn results in increased electrodermal amplitude [27]. Moreover, the intervention group had deceleration of HR, while HR of the sham and control groups remained unchanged. The effect of acupuncture on physiological parameters has been supported by pervious animal and human studies. Previous studies confirmed the influence of acupuncture on reduction of heart rate or pulse rate variability [28, 29]. As a possible mechanism, increased activity of parasympathetic nervous system and decreased activity of sympathetic nervous system might be accompanied by a decline in physiological stress symptoms and a subsequent reduction in anxiety [30]. Furthermore, Vickland et al. examining the influence of acupuncture on HR variability showed that anxiety could change the response of individuals to acupuncture. They revealed that acupuncture had a dominant parasympathetic effect only in individuals with lower level of anxiety [31]. They argued that anxiety might be treated with acupuncture but it could also affect the results of acupuncture directed at other symptoms such as HR variability. Indeed current data provided further support for the role of acupuncture in control and decrement of anxiety by modifying physiological outcomes.

Our results showed that the acupuncture led to significantly lower somatic and cognitive anxiety in contrast with sham and control conditions. This finding is in accordance with Boucher et al.'s study that indicated lower level of anxiety in seven college students who received TCM acupuncture treatment compared to sham group [32]. Studies demonstrated that participants who received acupuncture at “relaxation” point had a reduction in anxiety level [33, 34]. A recent systematic review supported the effectiveness of acupuncture as an alternative treatment for generalized anxiety [35]. However, acupuncture intervention had no specific influence on self-confidence of young athletes in current study. It seems that self-confidence of athletes remains stable in a week leading to a competition event [36]. Wiggins examined the pattern of anxiety and self-confidence in 24 hours, 2 hours, and 1 hour leading to a competition [10]. Their results demonstrated an increased somatic anxiety from 24 hours to 1 hour, though the intensity of cognitive anxiety and self-confidence remained stable. Based on these results, our a priori hypothesis that acupuncture could reduce state anxiety of athlete prior to a competition, though self-confidence of athletes might not be affected by an acupuncture intervention, is confirmed.

Our data is comparable with previous findings on efficacy of cognitive or behavioral interventions on competitive anxiety in athletes. Although there is no existing study simultaneously examining acupuncture and other cognitive or behavioral treatments for competitive anxiety, to provide a base case for comparison, we presented a few examples from anxiety interventions in athletes. Routhan and Ruhela indicated the effectiveness of chanting and music on decreasing competitive anxiety [37]. Chanting may have a few positive physiological or psychological effects, but it requires particular space to practice and relies on athlete expertise; also its supporting evidence is still moderate. Furthermore, to reduce anxiety in competition conditions, relaxation techniques may produce modest, short-term benefits but they are not as effective as comprehensive psychological treatments [38]. The durability of anxiolytic effects is an important issue in behavioral techniques such as relaxation. More recently CBTs which include both behavioral and cognitive strategies have been more frequently used to decrease athletes' competitive anxiety [13]. However, their usability may be hampered by some shortcomings such as time-consuming procedures and dependency on an expert counselor.

As far as we know, our study is the first of its kind which provided evidence to support the significance of the use of acupuncture in management of athletes' anxiety while warranting examining acupuncture in parallel with cognitive and behavioral treatments in future studies.

However, there were a few limitations in this study which should be noted. First, to examine different dimensions of competition anxiety, using a fine grained measurement seems more precise. It is important to measure intensity, frequency, and direction of competition anxiety separately. For example, assessing direction of athletes' anxiety helps the researchers to realize that athletes perceive their anxiety, facilitative or debilitative toward their performance [39]. Second, we administered acupuncture in both “Shen Men” and “relaxation” points to reduce anxiety. Thus, it is recommended to clarify whether stimulation “Shen Men” or “relaxation” point can result in reduction of anxiety. Another limitation that warranted further attention is about the assessment of possible comorbid mood disorders in athletes enrolled in acupuncture interventions. Future studies are recommended to screen depressive symptoms via valid assessment tools (e.g., general health questionnaire). Although it was unlikely that this population was affected by psychiatric disorders, we completed a clinical observation from athletes to exclude those with any psychiatric disorders including depressive disorders. Finally, to reduce the effect of possible confounders, medication use was defined as an exclusion criterion and participants were instructed not to use alcohol or caffeine and get a good night of rest 24 h before the assessment. However, we recommended future researchers to make exact assessment of possible confounders (e.g., physical activity) which may influence the association of acupuncture on psychological/physiological outcomes in young athletes.

In conclusion, the results showed that acupuncture is effective in modifying the physiological changes associated with skin conductance and heart rate and helps adolescents to decrease their anxiety prior to competition.

## Figures and Tables

**Table 1:** Physiological responses (skin conductance) for athletes in pre- and postintervention.

	Pretest (*μ*s)	Posttest (*μ*s)
Group 1, acupuncture	2.63 (0.64)	1.70 (0.63)
Group 2, sham	2.56 (0.60)	2.34 (0.56)
Group 3, control	2.60 (0.57)	2.48 (0.58)

**Table 2:** Physiological responses (heart rate) for athletes in pre- and postintervention.

	Pretest (bpm)	Posttest (bpm)
Group 1, acupuncture	65.5 (6.2)	62.9 (6.0)
Group 2, sham	63.8 (5.9)	64.2 (6.4)
Group 3, control	66.1 (6.9)	66.8 (6.3)

**Table 3:** Competitive state anxiety for athletes in pre- and postintervention.

Anxiety subscales	Group	Pretest	Posttest
Cognitive anxiety	Group 1, acupuncture	21.5 (5.3)	15.9 (4.8)
	Group 2, sham	22.1 (5.4)	19.6 (5.8)
	Group 3, control	21.9 (4.9)	21.2 (5.3)

Somatic anxiety	Group 1, acupuncture	20.8 (4.6)	13.7 (4.6)
	Group 2, sham	19.9 (4.8)	18.7 (5.0)
	Group 3, control	20.0 (5.1)	19.8 (4.5)

Self-confidence	Group 1, acupuncture	23.9 (5.6)	23.4 (5.5)
	Group 2, sham	20.0 (5.1)	19.8 (4.5)
	Group 3, control	22.8 (5.0)	21.9 (5.1)
